# Integrating a mental health intervention into PrEP services for South African young women: a human‐centred implementation research approach to intervention development

**DOI:** 10.1002/jia2.26274

**Published:** 2024-07-05

**Authors:** Jennifer Velloza, Nomhle Ndimande‐Khoza, Lisa Mills, Tessa Concepcion, Sanele Gumede, Hlukelo Chauke, Ruth Verhey, Dixon Chibanda, Sybil Hosek, Bryan J. Weiner, Connie Celum, Sinead Delany‐Moretlwe

**Affiliations:** ^1^ Department of Epidemiology & Biostatistics University of California San Francisco San Francisco California USA; ^2^ Wits RHI University of the Witwatersrand Johannesburg South Africa; ^3^ Department of Global Health University of Washington Seattle Washington USA; ^4^ Friendship Bench Program Harare Zimbabwe; ^5^ Department of Medicine University of Illinois Chicago Chicago Illinois USA; ^6^ Department of Medicine University of Washington Seattle Washington USA; ^7^ Department of Epidemiology University of Washington Seattle Washington USA

**Keywords:** HIV, pre‐exposure prophylaxis, mental health, adolescent girls and young women, human‐centred design, implementation science

## Abstract

**Introduction:**

Adolescent girls and young women (AGYW) who may benefit from HIV pre‐exposure prophylaxis (PrEP) face high levels of common mental disorders (e.g. depression, anxiety). Common mental disorders can reduce PrEP adherence and increase HIV risk, yet mental health interventions have not been well‐integrated into PrEP delivery.

**Methods:**

We conducted a four‐phase human‐centred design process, from December 2020 to April 2022, to understand mental health challenges among AGYW in Johannesburg, South Africa and barriers to integrated mental health and PrEP services. In the “Discover” phase, we conducted in‐depth interviews with AGYW and key informants (KIs) in Johannesburg. We conducted a rapid qualitative analysis, informed by the Consolidated Framework for Implementation Research (CFIR), to identify facilitators and barriers of integrated mental health and PrEP services and mapped barriers to potential implementation strategies. In the “Design” and “Build” phases, we conducted stakeholder workshops to iteratively adapt an evidence‐based mental health intervention, the Friendship Bench, and refine implementation strategies for South African PrEP delivery settings. In the “Test” phase, we piloted our adapted Friendship Bench package.

**Results:**

Interviews with 70 Discover phase participants (48 AGYW, 22 KIs) revealed the importance of integrated mental health and PrEP services for South African AGYW. Interviewees described barriers and implementation strategies for mental health and PrEP services around the CFIR domains: intervention characteristics (e.g. challenges with AGYW “opening up”); outer Johannesburg setting (e.g. community stigma); inner clinic setting (e.g. judgemental healthcare providers); characteristics of counsellors (e.g. training gaps); and the implementation process (e.g. need for demand creation). The Design and Build workshops included 13 AGYW and 15 KIs. Implementation barriers related to the quality and accessibility of public‐sector clinic services, lay counsellor training, and community education and demand creation activities were prioritized. This led to 12 key Friendship Bench adaptations and the specification of 10 implementation strategies that were acceptable and feasible in initial pilot testing with three AGYW.

**Conclusions:**

Using a human‐centred approach, we identified determinants and potential solutions for integrating mental health interventions within PrEP services for South African AGYW. This design process centred stakeholders’ perspectives, enabling rapid development of an adapted Friendship Bench intervention implementation package.

## 1 INTRODUCTION

African adolescent girls and young women (AGYW), ages 18–25 years, face HIV incidence rates of 4–7 per 100 person years [1, 2, 3]. Adolescence is a critical time for the onset of common mental disorders (CMDs) including depression and anxiety [4]. HIV incidence is higher among individuals living with CMD, and CMD symptoms can reduce engagement in HIV prevention and treatment cascades [4, 5, 6, 7, 8, 9]. Given the high HIV incidence and frequency of CMD (22%) among South African AGYW, effective interventions that address both health outcomes are needed [10].

Daily oral pre‐exposure prophylaxis (PrEP) is >90% effective at preventing HIV [11, 12, 13, 14]. However, AGYW in South Africa report barriers to oral PrEP adherence, including CMD symptoms [15, 16, 17, 18, 19]. Approximately 20–50% of South African AGYW initiating PrEP have mild‐to‐moderate CMD symptoms, often due to social, relationship and/or socio‐economic factors [17, 20]. CMD symptoms can persist for about 1 year in 49% of AGYW and result in maladaptive coping [17, 20]. Critically, those with CMD symptoms are 25% less likely to adhere to PrEP [17, 20]. Interventions that address CMD as part of PrEP adherence support may produce multiple health benefits by addressing individual emotions, promoting resiliency and improving coping.

Cognitive behavioural and problem‐solving therapies (CBT, PST) are effective treatments for mild‐to‐moderate CMD symptoms [21, 22, 23]. However, there is a large unmet need for coverage of these interventions [24, 25]. CBT and PST focus on teaching clients to identify problems and enact solutions, thus empowering them to solve modifiable issues while improving coping behaviours around less modifiable factors [26, 27, 28]. The Friendship Bench is one effective CBT and PST intervention shown to reduce depression among adults in Zimbabwean primary care clinics [29]. Core components include: CMD screening; 4–6 problem‐solving sessions delivered by a lay counsellor (with six steps of “opening up the mind” or inviting the clients to talk, setting goals, defining a top problem, brainstorming solutions, selecting solutions and developing an action plan); group sessions focused on economic empowerment; and lay counsellor training [29, 30, 31]. The first session generally includes all six steps and subsequent sessions focus on reviewing whether the action plan has worked and identifying other problems, as needed. CBT and PST improve antiretroviral therapy (ART) adherence in young women living with HIV [4, 32–34]. However, little prior research has been done to adapt CBT or PST for integration into PrEP delivery for AGYW or to explore challenges with implementation (e.g. fidelity, retention) [35]. We hypothesized that PrEP service delivery integrated with core components of the Friendship Bench would be seen as necessary and acceptable for South African AGYW but tailored modifications to the Friendship Bench's adaptable components (related to intervention content and implementation) would be needed to promote its use in PrEP delivery settings [29, 30, 31].

We used a human‐centred design (HCD) approach to modify the adaptable components of the Friendship Bench to optimize it for South African AGYW in PrEP delivery settings. HCD has been used to adapt mental health intervention content and implementation approaches through participatory, iterative stages of idea generation, prototype development and piloting [36]. We involved key stakeholders in co‐designing intervention components and implementation strategies to ensure they are acceptable and feasible for incorporating a mental health intervention into PrEP delivery in the South African context.

## 2 METHODS

**Figure 1:**
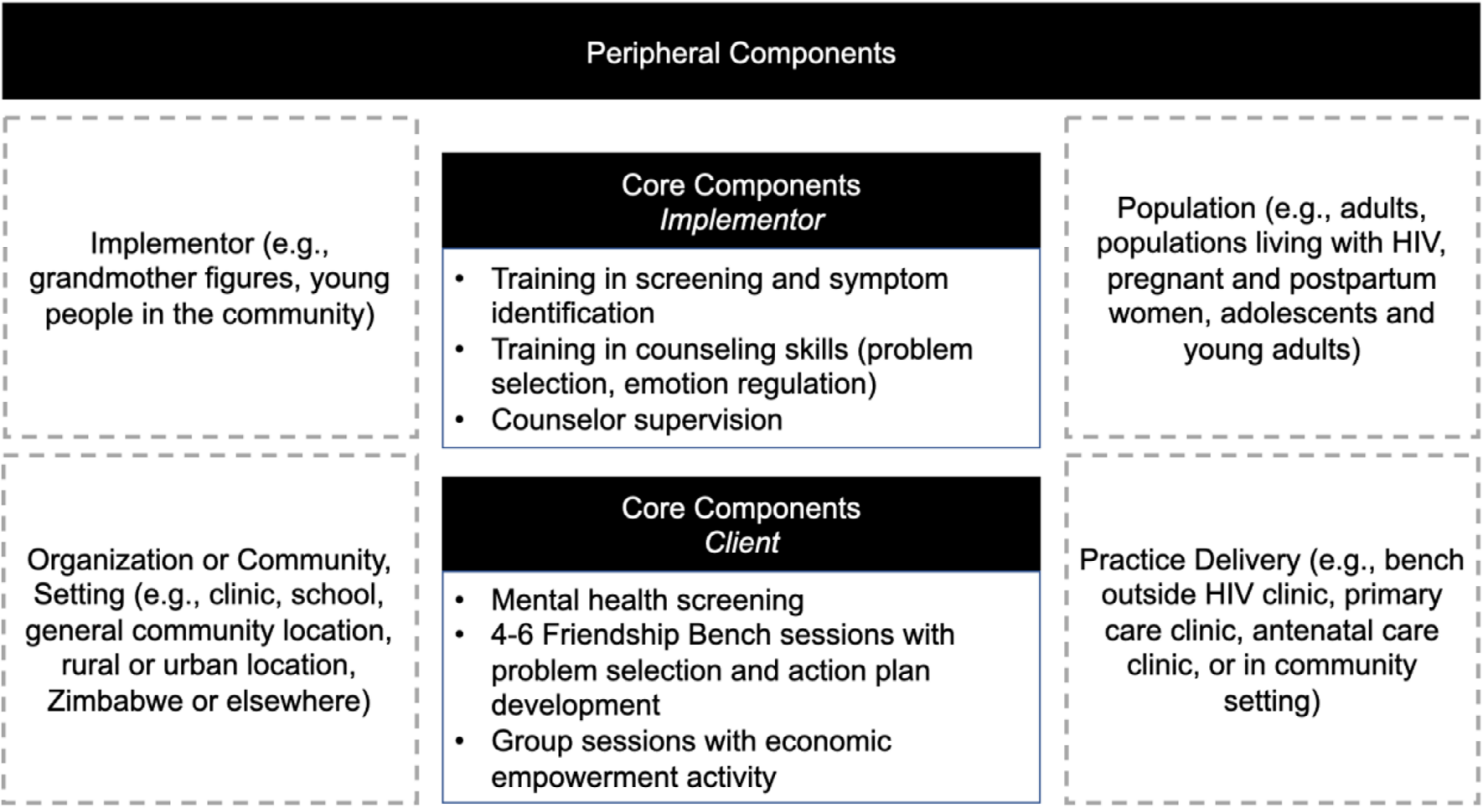
**Friendship Bench intervention core components and adaptable periphery**.

Adaptable components of the intervention and its implementation, identified through reviewing Friendship Bench literature and conversations with Friendship Bench developers, include decisions about the target population, who provides the counselling, and delivery setting (Figure 1). Our HCD work was focused on these components.

### 2.1 Study design

**Figure 2:**
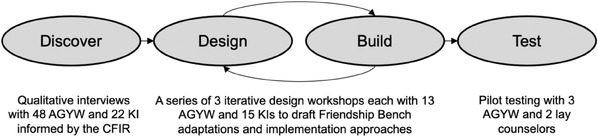
**Human‐centred design process guided by the Discover‐Design‐Build‐Test framework**. Abbreviations: AGYW, adolescent girls and young women; CFIR, Consolidated Framework for Implementation Research; KI, key informant.

Our HCD study was grounded in the “Discover‐Design‐Build‐Test” framework (Figure 2) [36]. The protocol was approved by ethics committees at the University of Witwatersrand and the University of Washington. Participants provided written informed consent in their preferred language. This study was conducted in Johannesburg, South Africa, from December 2020 to April 2022 (see Supporting Information for information on our team).

### 2.2 Discover Phase: study population, data collection and analysis

During the “Discover” phase (December 2020–December 2021), AGYW and stakeholders described needs and contextual factors that might affect intervention success. We conducted in‐depth interviews with AGYW in an ongoing PrEP programme [37], purposively sampled to include varying PrEP adherence (determined by tenofovir diphosphate concentrations [37]), depressive symptom severity (using the 10‐item Center for Epidemiologic Studies‐Depression [CES‐D] scale) and age. All were female at birth, 18–25 years old and had experience taking oral PrEP. Staff approached eligible AGYW to describe this study and obtain consent. We also interviewed key informants (KIs) who had experience with PrEP implementation or mental health service delivery in public clinics or related policy and/or programmes in South Africa. All KIs were identified by the team based on knowledge of the PrEP and mental health service delivery landscape and were approached in person or via phone or email.

Interview guides were informed by the Consolidated Framework for Implementation Research (CFIR), to ensure consideration around and organization of individual‐ and contextual‐level constructs that could influence intervention implementation [38]. Guides included questions about: perceptions of the Friendship Bench (“intervention” domain of the CFIR); norms, policies and structures that could impact integrated mental health and PrEP services (“outer setting”); mental health and PrEP service delivery in clinics (“inner setting”); training and experience to deliver the intervention and AGYW's needs around mental health and PrEP services (“individuals involved”); and implementation approaches for integrated services (“implementation process”) [38]. Participants did not have direct experience with the Friendship Bench prior to interviews, but we described the intervention core components using a structured script.

Interviews were conducted in English, isiZulu, SeSotho or Xitsonga and were recorded, transcribed and translated into English. We used a rapid qualitative analysis approach (developed by Hamilton) to identify facilitators and barriers to integrated mental health and PrEP service delivery and potential implementation strategies to overcome barriers [39, 40, 41]. First, experienced qualitative researchers (JV, NNK, LM, TC, SG and HC) created a summary sheet template deductively based on guides. The template included brief domain names corresponding to interview guide questions. We piloted the template with two transcripts to ensure reliability across researchers. Second, transcripts were divided between researchers and each summarized interview findings according to summary sheet domains. Third, we developed matrices to organize themes, whereby rows were participants and columns were summary sheet domains. Fourth, we extracted themes by reviewing matrix data within each column. Fifth, we mapped our key themes onto CFIR domains, using colour coding within our matrix to designate facilitators and barriers related to the five domains. We organized data on participant‐suggested implementation strategies that could address each of the barriers based on the CFIR‐Expert Recommendations for Implementing Change (ERIC) matching tool [42, 43]. The CFIR‐ERIC tool ensured that our implementation strategies were matched to barriers based on guidance from the implementation science field, grouped into evidence‐based categories, and comparable with other implementation studies.

### 2.3 Design and Build Phases: study population, data collection and analysis

During the “Design” and “Build” phases, AGYW and KIs iteratively developed and refined intervention adaptations and implementation strategy prototypes during a series of workshops. Workshops enrolled: AGYW who were female at birth, ages 18–25 and had experience taking PrEP (from completed studies or local clinics); AGYW ages 18–25 years from a Youth Community Advisory Board (YCAB) affiliated with the Wits Reproductive Health Institute (WRHI); and KIs recruited using similar means as in the “Discover” phase.

We conducted three, 3‐hour workshops with AGYW and KIs separately. The first and second workshops were conducted over two sequential days and the third occurred 1 week later to allow the team time to revise materials based on feedback. The same participants were invited to attend each of the workshops. Workshops were held over Zoom and participants were compensated for data usage. They were conducted in English but staff translated conversations in isiZulu as needed (translations were offered after English phrases were spoken). Each workshop was recorded and transcribed, and participants completed structured feedback forms after each.

During the first workshop, researchers presented information about the Friendship Bench, shared “Discover” phase findings, and explored potential implementation determinants and strategies for integrated Friendship Bench and PrEP delivery through large and small group activities. Miro^©^ software (2023, RealTimeBoard, Inc.) facilitated interactive responses to “How might we…?” questions developed based on the literature and our “Discover” findings (e.g. “How might we support staff to deliver the Friendship Bench?”). Participants were shown a list of implementation barriers identified in the “Discover” phase and asked to rank barriers by perceived strength of influence using a Likert scale (with 1 being the highest likelihood of influence). The top five ranked barriers were prioritized for intervention adaptation and implementation strategy development in the next set of workshop activities. Participants were asked to rank intervention adaptations and other implementation strategies identified in the “Discover” phase by perceived likelihood of success and feasibility based on their experiences and hypothetical scenarios [42]. Scores were on a 5‐point Likert scales, with 1 being the highest likelihood of success or feasibility. We calculated mean scores.

During the second workshop, participants selected and specified 1–2 intervention adaptations and other implementation strategies to address each of the top five implementation barriers [44]. While the CFIR‐ERIC matching tool enabled us to match implementation barriers with evidence‐based implementation strategies, these ERIC strategies are quite broad and this workshop focused on further specifying and tailoring the strategies to our context. The first two workshops were audio‐recorded, transcribed, and translated and our team reviewed the content and wrote analytic memos summarizing themes around intervention adaptations and implementation strategies to inform the third workshop. We used affinity mapping to group possible implementation strategies and intervention adaptations and sort them by each prioritized barrier, prior to developing rough prototypes of the top five implementation strategies and intervention adaptations.

At the third workshop, participants reviewed and provided feedback on prototypes of adaptable components of Friendship Bench intervention content, training manuals and demand creation materials. We again audio‐recorded, transcribed, and translated this workshop and wrote analytic memos summarizing participant feedback incorporation into the prototypes prior to the “Test” phase.

### 2.4 Test phase: study population, data collection and adaptation documentation

The goal of the “Test” phase goal was to conduct a brief, small‐scale pilot to understand user experiences and confirm the acceptability and feasibility of intervention components, in a real‐world context, to inform a larger‐scale study [36]. This phase included AGYW who were female at birth, ages 18–25, with experience taking PrEP and mild‐to‐moderate symptoms of CMD, as determined by a score ≥7 on the Self Reporting Questionnaire 20‐item (SRQ‐20) [45]. All participants were literate in English and/or isiZulu.

Following workshop completion, we trained two female lay counsellors in the adapted Friendship Bench intervention. The lay counsellors were near peers and did not have prior university‐level training in psychology or counselling. Each participant completed one mock counselling session (which was limited to the content covered in the first Youth Friendship Bench SA session and did not include other implementation strategies). All sessions were conducted in person and recorded. Participants and counsellors completed post‐session debriefing reports. Counsellors also completed Zoom debrief sessions with the team. Revisions were made based on the strength of the recommendations and feasibility of implementation, and documented using the Framework for Reporting Adaptation and Modifications (FRAME) [46].

## 3 RESULTS

Across all phases of our work, we enrolled 64 AGYW and 37 KIs including interviews with 48 AGYW and 22 KIs in the “Discover” phase, workshops with 13 AGYW and 15 KIs in the “Design” and “Build” phases, and pilot testing with three AGYW in the “Test” phase.

### 3.1 “Discover”: intervention acceptability and determinants of intervention delivery

AGYW participants were on average 21 years, 24 (50%) had CES‐D scores ≥10 and 20 (42%) were adherent to PrEP (Table S1). Fifteen (68%) KIs were female and 12 (55%) were PrEP providers. The “Discover” phase revealed high demand for mental health services and acceptability of Friendship Bench integration within PrEP delivery. Acceptability was driven by the perceived burden of CMD among AGYW, a lack of resources for addressing CMD symptoms and beliefs that a mental health intervention could improve CMD and PrEP use for AGYW:

“I overthink a lot. That's why I have anxiety, even depression. Having someone to talk to is a great idea. You could be surprised by how may people are going through depression especially because of their relationships.” *AGYW, 19 years, low PrEP adherence*

AGYW identified that CMD symptoms were often precipitated by concerns about relationships, food and financial insecurity, and a lack of support in the community to address mental health and wellbeing:

“You might not understand what I'm going through because you've never been there. I cannot come talk to you and say I was raped. We need someone who knows how difficult it is for a black child to get a job out there, or go to school with an empty stomach.” *AGYW, 24 years, low PrEP adherence*

KIs also acknowledged that “mental health is a huge issue” and noted that symptoms of CMD appeared to be linked to “gender‐based violence (GBV) and negative situations around us.” All participants were optimistic that an intervention to address mental health among AGYW could improve empowerment, PrEP use and overall wellbeing:

“Young people are finding themselves. They are going through a lot of things, they've got pressures from peers, school…some of them might also experience abusive relationship, maybe get pressure from parents because drugs we are using for PrEP are the same drugs used for [HIV treatment]…So it's very important for us to give them tools on how to handle pressures that come with everything that pertains to [HIV prevention] but for life in general.” *Key informant, project manager for PrEP delivery programme*

Table 1 summarizes findings from the “Discover” phase according to the five CFIR domains regarding facilitators (green cells in Table 1) and barriers (yellow cells) to integrated mental health and PrEP service delivery, as well as possible implementation strategies to address these barriers.

**Table 1:** Summary of key themes from the “Discover” qualitative phase, organized by CFIR domains and participant type^a^

CFIR domain	Implementation determinants identified by AGYW	Implementation determinants identified by staff and key informants	Potential implementation strategies
Characteristics of the intervention—*psychosocial counselling*		Building capacity for psychosocial counselling is more appealing than medication and standard‐of‐care referrals	Refined screening practices to triage AGYW *(Staff, KI)* Person‐centred, stepped care models tailored to AGYW needs *(Staff, KI)* Warm hand‐offs (ensuring connection) for all referral services *(Staff, KI)* Adapt intervention to incorporate local perspectives and content relevant for AGYW *(AGYW, Staff, KI)* Adapt clinic flow to maximize time efficiency for AGYW *(AGYW, Staff, KI)*
	AGYW may be reluctant to open up, especially in face‐to‐face interaction or if they have to travel to clinic and wait	Unethical to screen for a counselling intervention if no specialists are available for referral; could result in long clinic wait times	
Outer setting—*urban Johannesburg, South Africa*	Clear need for psychotherapy related to depression and other mental health issues (gender‐based violence, relationship issues, PrEP stigma)	Mental health service integration aligns with South African Department of Health goals	Community outreach activities for demand‐creation, PrEP and mental health stigma reduction *(AGYW, Staff, KI)* Conduct group sessions with AGYW to normalize mental health issues *(AGYW, Staff, KI)* Inventory referral services and engage key stakeholders to identify a broad range of referral options to address mental health issues and gender‐based violence *(Staff, KI)*
	Community stigma around mental health awareness and care‐seeking	Community stigma around mental health awareness and care‐seeking; lack of clarity on referral options and lack of existing services	
Inner setting—*Ward 21 adolescent‐friendly PrEP clinic*		Strong network to support within‐clinic referrals; some mental health services already provided (referrals, some screening) although without formal procedures or monitoring	Create a non‐judgemental and friendly atmosphere for AGYW *(AGYW, Staff, KI)* Incorporate mental health screening and counselling with other youth‐friendly services (e.g. PrEP counselling) to maximize efficiency *(Staff, KI)* Create a rewards system for counsellors with recognition for outcomes *(Staff, KI)* Training, supervision and peer support to empower counsellors *(Staff, KI)* Engage clinic leadership to “own” the intervention *(Staff, KI)*
	Provider judgement and discrimination among staff; long clinic wait times to see providers and counsellors	Provider judgement and discrimination among staff; tension between wanting to provide psychotherapy but also not adding more staff work or clinic inefficiencies; lack of time, space and funding for counsellors	
Characteristics of individuals involved—*lay counsellors*	Judgemental attitudes among providers	Judgemental attitudes among providers; lack of counsellor training on mental health and psychotherapy approaches	Train counsellors in adolescent‐friendly communication skills and psychotherapy counselling approaches *(AGYW, Staff, KI)* Conduct training on patient‐counsellor confidentiality *(AGYW, Staff, KI)* Provide a space for counsellors to process difficult cases *(Staff, KI)* Regular supervision with audit and feedback to promote counsellor self‐efficacy for intervention delivery *(Staff, KI)*
Process	Normalizing mental health challenges among peers and in the community	External change agents (celebrities, social media influencers) have spoken out about mental health during COVID	Engage AGYW as peer champions and social influencers to conduct demand creation *(AGYW, Staff, KI)*

Abbreviations: AGYW, adolescent girls and young women; CFIR, Consolidated Framework for Implementation Research; KI, key informant; PrEP, pre‐exposure prophylaxis.

^a^ Themes representing potential facilitators of mental health and PrEP service integration are shaded in green, while themes representing potential barriers are shaded in yellow. Blank cells are those where no determinant was mentioned for that CFIR domain and participant type.

#### 3.1.1 Characteristics of the intervention

Participants raised concerns about Friendship Bench components. AGYW worried about a potential lack of trust in providers and AGYW willingness to open up in face‐to‐face interactions. They also identified concerns about frequent clinic visits to attend 4–6 prescribed sessions. KIs had similar concerns about frequent visits and hesitations about screening for mental health conditions if referral pathways were unclear.

Several potential implementation strategies suggested to mitigate these concerns included: screening to triage AGYW for additional services; stepped care for those needing additional support; tailoring the Friendship Bench to AGYW developmental needs and local context; and an adapted clinic flow to maximize efficiency.

#### 3.1.2 Outer setting

Participants described community stigma around mental health disorders and care‐seeking as barriers to intervention delivery:

“It's the stigma associated with somebody seeking out that sort of counselling—that's the main thing is to be wary of.” *Key informant, psychologist and mental healthcare lead at local clinic*

Clinic‐based KI articulated a lack of information about existing community resources for mental healthcare. KIs argued that Friendship Bench delivery would be facilitated by alignment between this intervention and government priorities for mental health service integration.

Proposed implementation strategies to address outer setting barriers included: inventory of existing referral services to ensure adequate resources are available to address AGYW's mental health needs; stigma reduction activities; and group sessions with AGYW to normalize health‐seeking behaviour for mental health.

#### 3.1.3 Inner setting

All participants were concerned about experiencing judgement from providers in public clinics in Johannesburg, given that both HIV and mental health conditions are highly stigmatized:

“Woahhhh, so you want this to be done in public clinics. That's going to be weird cause most of the time, young ladies feel like they're judged in public clinics…They would be like, ‘if I had to raise something like this to a nurse in a public clinic, you get some backlash’.” *Clinic staff member*

KIs were also concerned about long clinic wait times due to a lack of provider availability, clinic space and funding. Clinic‐based KI described a tension between the importance of providing psychotherapy for AGYW and not wanting to create additional work for providers. AGYW described a desire to locate Friendship Bench delivery within the clinic (as long as services could be provided by trained lay counsellors rather than their usual providers), instead of a community setting. This clinic‐based approach was described as potentially reducing stigma related to being seen seeking mental health‐specific services and facilitating integrated service delivery for AGYW who may be already visiting the clinic for other healthcare.

Potential inner‐setting implementation strategies include: integrating mental health screening and supportive counselling within PrEP counselling to maximize efficiencies; conducting counselling in an adolescent‐friendly clinic space; training, supervising and rewarding peer counsellors for their work; and engaging clinic leadership to “own” the intervention. AGYW also described the need to create a non‐judgemental and friendly clinic atmosphere to reduce stigma.

#### 3.1.4 Characteristics of individuals involved

Provider attributes including lack of training on mental health and psychotherapy approaches and judgemental attitudes were key barriers to Friendship Bench integration into PrEP delivery. No facilitators were mentioned. Implementation strategies discussed included: training on adolescent‐friendly communication, psychotherapy and confidentiality; and supportive supervision for counsellors to mitigate vicarious trauma that could be triggered by patients’ experiences. AGYW highlighted the need not to feel pathologized but to have relatable counsellors:

“Just having a friendly conversation rather than trying to diagnose what is wrong with our mental health…You just talk to them in a sense that they would understand where you're coming from.” *AGYW, 19 years, low PrEP adherence*

#### 3.1.5 Implementation process

Two facilitators of the implementation process were discussed, namely recent cultural shifts to destigmatize mental health challenges in the community and the presence of influential individuals speaking openly about mental health in South Africa. An implementation strategy to magnify these facilitators included engaging AGYW as peer champions and “social influencers” to conduct demand creation around mental health and PrEP programmes:

“The important thing is that it should be offered by young people themselves…I think it's more easy for [AGYW] to relate to [other young people] because they speak the same language.” *Key informant, project manager for PrEP delivery programme*

### 3.2 “Design” and “Build”: barrier prioritization and implementation strategy specification

For the “Design” and “Build” workshops, seven AGYW (54%) had experience receiving PrEP and 46% were members of the local YCAB. All KIs were female, and about half were research clinic staff (*N* = 8, 53%), while half were involved in PrEP or mental health implementation in public clinics, policy and/or funding (*N* = 7, 47%). A total of eight (53%) had a position focused on mental health research or services (e.g. psychologist, technical advisor on mental health programming).

At the start of the workshops, AGYW and KIs reviewed themes from the “Discover” phase and agreed with all barriers and strategies presented. AGYW and KIs also shared their own perspectives on implementation barriers and strategies, building off “Discover” phase findings and identified several new barriers as indicated below. During their first workshop, AGYW described 36 implementation barriers to Friendship Bench integration with PrEP. After working in small groups to answer the question, *“Which do you think could pose the biggest problems to successful implementation?”* AGYW identified five priority barriers from the larger list: (1) stigma around mental healthcare and PrEP; (2) concerns about poor service at the clinic including breaches of confidentiality; (3) lengthy clinic wait times and large travel distances to clinic; (4) trust issues with parents and partners (mentioned for the first time during the workshops and not part of “Discover” phase findings); and (5) consistency in attending sessions (Table 2; Table S2 for representative quotations).

**Table 2:** Summary of AGYW and KI workshop findings on implementation barriers and implementation strategies

Implementation barrier domain^a^	Theme	ERIC implementation strategy category	Description of specified implementation strategy^b^
***AGYW Workshop Findings***			
Judgement taking PrEP or seeking mental healthcare	There is judgement and stigma around PrEP and mental health service seeking	Develop educational materials; distribute educational materials; use mass media	Community education via social media and television advertisements to share with the community the benefits of taking PrEP and participating in a mental health intervention
Poor service	AGYW feel they are mistreated, ignored or disrespected and are concerned about confidentiality	Audit and provide feedback; provide clinical supervision	Counsellor monitoring and management to help hold staff accountable, improve their skills and make management more visible to AGYW
Time spent at clinic	AGYW do not want to spend a lot of time at the clinic or travel a large distance to get there	Create new clinical teams; revise professional roles	Increase staffing capacity
Trust issues	Parents and partners will be concerned about PrEP use and mental health issues	Inform local opinion leaders; involve patients and family members	Hold community meetings at schools to talk about PrEP and mental health issues; allow partners to accompany one another at the clinic
Consistency of participation	AGYW may start sessions but will not continue with sessions regularly	Intervene with patients to enhance uptake and adherence	Empowering SMS reminder messages to encourage AGYW to keep coming to the clinic and engaging with PrEP and mental healthcare
***KI Workshop Findings***			
Accessibility	It is important to ensure appropriate location for the bench itself and to help make the clinic more accessible for AGYW; will need to ensure confidentiality and safety on the bench	Change service sites	Create an option for a virtual Friendship Bench to make the service tailored to AGYW needs, if they would prefer a physical bench or a phone call
Staff knowledge and ability	Counsellors may not know where to refer patients and will not know about mental health counselling or the Friendship Bench	Provide clinical supervision; centralize technical assistance; create a learning collaborative	Set up WhatsApp groups for supervision and debriefing
Stigma	There is a large amount of community, family and facility‐level stigma related to PrEP, HIV and mental health issues	Identify and prepare champions; inform local opinion leaders	Engage community stakeholders (e.g. key celebrities, gatekeepers, school administrators) in creating a dialogue around mental health awareness
Resources	Clinics are lacking staff (including counsellors, social workers), referral points outside the clinic and screening tools	Change physical structure and equipment	Use a mobile, digitized self‐screening tool (with a WiFi screening point) so that AGYW can screen themselves, which would reduce stigma and also help streamline clinic flow
AGYW needs	AGYW may not want to talk about mental health issues, they likely have other more pressing concerns (e.g. food insecurity), and do not want to spend a lot of time at the facility	Increase demand	Reframe mental health needs from a positive lens (e.g. use words like “well‐being” instead of “problems”)

Abbreviations: AGYW, adolescent girl and young women; ERIC, Expert Recommendations for Implementing Change; PrEP, pre‐exposure prophylaxis; SMS, short message service.

^a^ Barriers were ranked by perceived strength of the barrier to negatively impact integrated mental health and PrEP service delivery. Participants were asked to rank each barrier on a scale from 1 to 5, with 1 being the barrier they perceived to have the strongest influence on intervention delivery and 5 being the barrier they perceived to have the weakest influence on intervention delivery. AGYW workshop rankings were as follows: poor service = 1.7; judgement taking PrEP = 2.8; time spent at the clinic = 2.8; trust issues = 3.0; consistency of participation = 3.5. KI workshop rankings were as follows: accessibility = 2.3; staff knowledge and ability = 2.7; stigma = 3.0; resources = 3.0; AGYW needs = 4.0.

^b^ The implementation strategies shown are those that participants selected to specify during subsequent workshops. This was determined based on how likely the strategy would be to succeed and how feasible the strategy would be to implement. Participants were asked to provide Likert‐style responses on how likely a strategy would be to succeed (from 1: Strongly Agree to 4: Strongly Disagree) and how feasible a strategy would be to implement (from 1: Strongly Agree to 4: Strongly Disagree). From AGYW workshops, all selected strategies had high likelihood of success (range: 1.1–1.6) and feasibility (range: 1.6–1.9). From KI workshops, all selected strategies had high likelihood of success (range: 1.0–1.7) and feasibility (range: 1.3–2.3).

During brainstorming, AGYW identified 3–7 potential intervention adaptations and other implementation strategies for each of the five prioritized barriers. After ranking each strategy for its potential feasibility and likelihood of implementation success, one adaptation or implementation strategy was selected per barrier: (1) counsellor monitoring and supervision; (2) community education; (3) increasing staff capacity; (4) engaging local opinion leaders; and (5) sending SMS appointment reminder messages (Table 2).

Clinic KI described 21 discrete implementation barriers to integrated Friendship Bench and PrEP services. These barriers were similar to those described by the AGYW (e.g. concerns about clinic resources) and also included unique barriers such as concerns with provider buy‐in and sustainability of this integrated model. KI selected five top priority barriers: (1) accessibility of the clinic and Friendship Bench to AGYW; (2) staff knowledge and ability to deliver integrated care; (3) mental health and HIV‐related stigma; (4) resource shortages within the clinic and for external referrals; and (5) competing needs among AGYW (mentioned for the first time during the workshops and not part of “Discover” phase findings; Table 2; Table S2 for representative quotations).

KI identified 4–6 potential intervention adaptations or other implementation strategies for each prioritized barrier: (1) creating a “virtual Friendship Bench” for AGYW with difficulties reaching the clinic; (2) establishing WhatsApp groups for counsellors; (3) identifying community champions to create a dialogue around mental health awareness; (4) developing a mobile, digitized CMD symptom self‐screening tool using a validated tool like the SRQ‐20; and (5) conducting demand creation (Table 2).

We consolidated a list of prioritized barriers and intervention adaptations and implementation strategies from the AGYW and KI workshops (Table 3). We prototyped several “content” modifications during the third workshop day. Specifically, we developed recruitment language to reframe demand creation through an empowerment‐based lens (e.g. using “mental health and well‐being” instead of “mental health problems”). We also selected the SRQ‐20 as the CMD screening tool for this context, given its prior validation in South Africa and participants’ perspectives that it would accurately capture somatic and psychological experiences. Finally, we modified the Friendship Bench's economic empowerment group session to focus on university and job applications.

**Table 3:** Summary of Friendship Bench adaptations, classified according to the Framework for Reporting Adaptations and Modifications to Evidence‐Based Implementation Strategies (FRAME‐IS)

Adaptation	Type of modification	Nature of modification^a^	Primary goal(s)
Recruitment materials reframing mental health issues from an empowerment‐based lens	Content	Changes in packaging and materials related to recruitment and demand creation	Increase reach; increase acceptability and appropriateness; increase health equity
Screening using SRQ‐20, completed via CASI on a tablet in the waiting room^b^	Content	Changes in materials (screening tool) and packaging (self‐administration via a tablet application)	Increase adoption; increase sustainability; increase acceptability and appropriateness
Group session focused on university and job applications and financial resources	Content	Substituting the group session on basket‐weaving with group income‐generating activities relevant to the population	Increase clinical effectiveness
Offer option of phone sessions after first in person session	Context: format	N/A	Increase reach; increase clinical effectiveness; increase health equity
One‐way SMS reminder messages for AGYW	Context: format	N/A	Increase reach; increase clinical effectiveness
Employ young women to conduct counselling sessions^b^	Context: personnel	N/A	Increase acceptability and appropriateness
Offer option of bringing in parents and/or partners to a counselling session	Context: population	N/A	Increase reach; increase clinical effectiveness; increase acceptability and appropriateness
Community outreach and education with gatekeepers including parents, school teachers, religious leaders and other important adults in the community	Context: population	N/A	Increase reach, increase acceptability and appropriateness
Conduct sessions in a private clinic room^b^	Context: setting	N/A	Increase acceptability and appropriateness
Include additional counsellor training and resources on gender‐based violence, financial insecurity and employment concerns^b^	Training	Tailoring the content of the Friendship Bench training materials to fit the local population needs and context	Increase acceptability and appropriateness; increase clinical effectiveness
Revise materials to include local idioms of distress and relevant words for AGYW^b^	Training	Tailoring the content of the Friendship Bench training materials to fit the local population needs and context	Increase acceptability and appropriateness; increase reach
WhatsApp support and supervision groups for counsellors	Training	Adding an additional asynchronous, virtual approach to counsellor support and supervision	Improve fidelity

Abbreviations: AGYW, adolescent girls and young women; CASI, computer‐assisted self‐interviewing; SMS, short message service; SRQ‐20, self‐reporting questionnaire 20‐item.

^a^ This column is only relevant for content, evaluation or training modification.

^b^ These adaptations were piloted in our “Test” phase with two lay counsellors and three AGYW.

We also specified “context” and “training” adaptations for our implementation strategies (Table 3). For example, the “virtual Friendship Bench” would include 25–30 minute phone calls with lay counsellors every 2–4 weeks. Reminders to boost AGYW engagement would be one‐way supportive SMS messages sent through an automated programme 1 week prior to appointments. We revised lay counsellor training materials to include additional content on GBV and food or financial insecurity. We also developed a lay counsellor supervision plan including WhatsApp groups for asynchronous support, weekly meetings between the counsellors and a local mental health professional, and regular review of counselling audio‐recordings.

### 3.3 Test phase: intervention adaptations and pilot

We enrolled three AGYW in the “Test” phase, all of whom had previously taken PrEP as part of a trial. In Zoom debriefs with the team at the conclusion of each in‐person counselling session, our adaptations to the intervention content (limited to the materials for counsellor training and the first session and the use of the SRQ‐20 tool) were found to be acceptable and appropriate to AGYW and counsellors. In particular, counsellors appreciated: the SRQ‐20 to identify key symptoms to focus on during sessions; GBV and financial insecurity content which arose with pilot participants; and the revised counselling materials tailored to local idioms. AGYW felt heard and that their problems were adequately addressed after the sessions. No further adaptations were developed or modified as a result of Test phase findings.

## 4 DISCUSSION

We used an HCD approach to adapt the Friendship Bench and identify implementation strategies to inform a future trial. This HCD approach emphasized stakeholder inclusion to identify a potentially acceptable and feasible model for integrating an evidence‐based mental health intervention into PrEP delivery. Our findings led us to revise intervention content and specify multiple implementation strategies and contribute to a growing body of research on the use of HCD to advance intervention adaptation and implementation [36, 47].

“Discover” phase findings reveal high unmet need for mental health services among AGYW at risk of HIV. Implementation science studies exploring determinants of integrated mental health and HIV service delivery have also highlighted the lack of trained personnel with skills for delivering mental health services as a primary barrier to integrated care [48, 49]. Additional barriers described in the literature and similarly found in the “Discover” phase include limited mental health literacy, lack of clear referrals and lack of adolescent‐friendly service‐delivery spaces [48, 49]. Both adolescents and providers in Southern and Eastern Africa have also cited concerns about the double burden of poor mental health and HIV‐related stigma faced by AGYW, although evidence suggests that demand‐creation activities in conjunction with youth‐friendly mental health services could reduce individual‐ and community‐level stigma [49, 50].

Our “Discover” phase also highlighted potential implementation approaches to overcome barriers to mental health service integration within PrEP delivery for South African AGYW. Recent qualitative work with South African primary providers reported a similar set of potential implementation strategies to promote mental health services in primary care, including community outreach, staff supervision, flexible service delivery models, and screening and referral mechanisms [51, 52]. Studies to adapt the Friendship Bench in Botswana, Vietnam and Malawi have all reported challenges with training and supervising younger lay counsellors, intervention fidelity, loss to follow‐up and community stigma, underscoring the importance and relevance of the implementation approaches identified in our research [35, 53, 54, 55, 56]. Evidence for implementation strategies to improve the delivery of mental health interventions in resource‐constrained settings is increasing. For example, one study in Kenya piloted an intervention called “Inuka,” based on the Friendship Bench, and delivered the intervention via a mobile application staffed by lay volunteers; this approach was acceptable and effective at reducing symptoms of CMD [54]. Teams delivering the Friendship Bench in Botswana developed and tested a lay counsellor supervision model which includes on‐site professional support for counsellors and case review with mental health professionals [57]. We plan to test the implementation strategies identified in our HCD process in a subsequent trial, to build this evidence base on the acceptability and effectiveness of these approaches to promote mental health service delivery in resource‐constrained settings.

The use of the “Discover‐Design‐Build‐Test” framework, with accompanying implementation science frameworks, allowed us to develop a set of implementation strategies for the Friendship Bench through interdisciplinary elements of stakeholder collaboration, contextual understanding and co‐design. While other qualitative and participatory research approaches exist, HCD was the ideal fit for this study for its emphasis on rapid, iterative design and testing cycles and practical outcomes [36]. Traditional qualitative approaches (e.g. interviews, focus group discussions) may have yielded similar initial themes on needs around and preferences for mental health services, but our iterative design cycles allowed us to refine themes with participant input and thoroughly probe areas of group consensus and divergence to arrive at a consolidated and prioritized list of implementation barriers and strategies important to stakeholders. In addition, the HCD workshops allowed us to focus our qualitative inquiry on developing tangible products (e.g. counsellor manuals), whereas traditional qualitative methods would have produced themes around preferences for adaptations that the study team would then be responsible for interpreting and implementing. HCD methods have been used to design implementation strategies for perinatal [58], hypertension [59], cancer [60], HIV [61] and mental healthcare interventions [36, 62].

Our goal was to identify and prioritize determinants of a mental health intervention integrated into PrEP delivery, followed by matching specific implementation strategies to address identified barriers. Few published methods exist for implementation determinant prioritization and implementation strategy matching, although this is an area of growing interest for the implementation science field [63]. The CFIR‐ERIC matching tool is one approach to link implementation barriers with response strategies [43], but little empiric research has been done to assess which implementation strategy(s) may be best for each barrier. In addition, the implementation strategies highlighted in the ERIC compilation are broad and studies seeking to evaluate these strategies need to further “specify” them to continue to build evidence on how and when these strategies may work best [44]. While the use of the ERIC ensured that our implementation strategies aligned with the existing implementation science evidence base and could be potentially comparable with other studies, the general ERIC categories fail to convey the nuance of our rich discussions during the HCD workshops. To mitigate this limitation of the ERIC compilation, we worked with workshop participants to further specify and tailor selected implementation strategies for our study population and context and will continue to document ways in which these strategies are operationalized and modified in our future work [64]. While this practice of matching implementation barriers to strategies is still nascent, future studies will need to assess trade‐offs between using a deductive approach starting with a broad categorization approach like the CFIR‐ERIC tool, conducting a more inductive process led by stakeholder perspectives or using some combination of methods.

Strengths of this study include the use of a rigorous and iterative data collection process, grounded in theoretical framings from HCD and implementation science. We engaged participants across multiple stakeholder groups to elicit client and provider perspectives. We ensured that our HCD process was grounded in the principle of empathetic design by including study team members with extensive prior experience working with this community, involving a YCAB and meeting regularly as a team to ensure that our findings were directly translatable. To ensure the validity of our findings, we reviewed data with workshop participants during each session to test the emerging themes, compared themes to literature and regularly developed summary memos with all team members to reflect on key findings. While the sample sizes for the “Design” and “Build” workshops allowed us to promote high levels of participant engagement during the workshops, we only piloted the adapted intervention with two counsellors and three AGYW. The “Test” phase only focused on the first Youth Friendship Bench SA intervention session, because it generally is the longest and most critical session that sets the groundwork for subsequent sessions, but we did not pilot several implementation strategies (e.g. remote visits, SMS reminders) or other counselling session topics due to time constraints and difficulties simulating real‐world retention challenges in the context of a pilot. This was appropriate for rapid HCD piloting [36, 47], but more extensive field testing is needed to determine the acceptability and feasibility of our full intervention package alongside the implementation strategies in a larger sample. Workshops were conducted via Zoom, which ensured high retention but could have limited group cohesion and participants’ comfort sharing with one another. We conducted separate workshops with AGYW and KIs to account for power dynamics between these groups. However, this approach prevented us from discussing themes across groups which may have elicited different results. All workshops were conducted in English; however, some conversations around idioms of mental distress were better expressed in isiZulu. Workshop facilitators provided isiZulu translations of these words or phrases as situations of comprehension arose, but participants’ responses may have been affected by the translations provided and their comfort speaking in English. Workshops were conducted using a semi‐structured facilitation guide developed *a priori*. While there was space for participants to take the discussion in new or unexpected directions, we did not iteratively update the guide between workshops and this semi‐structured approach may have limited our findings to certain topics of conversation. Finally, participants reviewed summarized data from each prior workshop at the start of the next workshop but did not otherwise participate in the data analysis and consolidation process between workshops.

## 5 CONCLUSIONS

Our study highlights the utility of an HCD approach, paired with implementation science frameworks, to guide the adaptation of an evidence‐based mental health intervention for AGYW alongside the development of implementation strategies to support intervention integration within HIV prevention services in South Africa. We identified key adaptations to Friendship Bench intervention content and intervention implementation strategies for this setting and population. These findings emphasize the importance of including client and KI perspectives when designing interventions and implementation strategies, particularly for stigmatized conditions like mental health and HIV, to ensure success at the individual‐, clinic‐ and community‐levels. This work paves the way for future research to evaluate whether our model of integrated mental healthcare within PrEP delivery is feasible, acceptable and effective in reducing poor mental health and improving PrEP adherence among South African AGYW.

## COMPETING INTERESTS

The authors report no competing interests.

## AUTHORS’ CONTRIBUTIONS

JV, CC and SD‐M designed the study. JV, NN‐K, LM, TC, SG and HC conducted all analyses. JV wrote the manuscript. All authors reviewed and approved the final version of the manuscript. JV: Funding, study design, data analysis, results interpretation and manuscript first draft. NN‐K: Data analysis, results interpretation and edited manuscript. LM: Data collection, data analysis, results interpretation and edited manuscript. TC: Data analysis, results interpretation and edited manuscript. SG: Data collection, data analysis, results interpretation and edited manuscript. HC: Data collection, data analysis, results interpretation and edited manuscript. RV: Study design, results interpretation and edited manuscript. DC and SH: Edited manuscript. BJW: Study design and edited manuscript. CC and SD‐M: Study design, results interpretation and edited manuscript.

## FUNDING

This work was supported by award numbers K99 MH123369 and R00 MH123369 (PI: Velloza) and R01 MH114544 (MPI: Celum, Delany‐Moretlwe) from the National Institutes of Mental Health (NIMH) of the National Institutes of Health (NIH). Dr. Velloza also received mentored support through the HIV, Infectious Disease, and Global Health Implementation Research Institute at the Washington University in St. Louis (HIGH IRI; PIs: Geng, Brownson; Fellow: Velloza). The University of Washington BIRCH Center (P30 MH123248; PI: Simoni) provided methodological consultation services on implementation science frameworks, barrier and implementation strategy mapping approaches, and rapid qualitative analysis.

## DISCLAIMER

The content is solely the responsibility of the authors and does not necessarily represent the official views of the NIH.

## CME STATEMENT

This article is published as part of a supplement supported by unrestricted educational grant by ViiV Healthcare.

Credits Available for this Activity: American Medical Association (AMA Credit).

Washington University School of Medicine in St. Louis designates this enduring material for a maximum of 1 AMA PRA Category 1 Credit™. Physicians should claim only the credit commensurate with the extent of their participation in the activity.

## Supporting information


**Table S1**: Characteristics of the qualitative “Design” phase sample
**Table S2**: Representative quotations from AGYW and KI workshops on key implementation barriers

## Data Availability

The data that support the findings of this study are available from the corresponding author upon reasonable request.
