# Supplementary material for: Integrating a mental health intervention into PrEP services for South African young women: a human‐centred implementation research approach to intervention development
Source: J Int AIDS Soc. 2024 Jul 5;27(Suppl 1):e26274. doi: 10.1002/jia2.26274 (PMC11224591; doi:10.1002/jia2.26274)
Supplement: Supplementary file 1 — Table S1: Characteristics of the qualitative “Design” phase sample Table S2: Representative quotations from AGYW and KI workshops on key implementation barriers [file JIA2-27-e26274-s001.docx]

**Supplementary Material**

***Study Team Reflexivity Statement***

All phases included team members from Johannesburg and the United States, with training in social science research, epidemiology, psychology, and HIV care. The Johannesburg team members have been conducting studies with AGYW from 1-10 years and the research team members from the United States have also worked with this population in Johannesburg for over 5 years. The majority of team members were White, female, and of higher socioeconomic status than the general population; however, the “Discover” phase interviews and the “Test” phase piloting were led by Black South African researchers and lay counselors. The study team did not undergo formal reflexivity training prior to the start of this study which may have influenced interpretation of study findings; however, all had previously considered the influence of their positions and social identities on their work and our team included diverse members with a broad range of perspectives to ensure rigorous data analysis.

**Supplemental Table 1.** Characteristics of the qualitative “Design” phase sample^1^

| **Characteristic^2^** | **AGYW**  **(N=48)** | **Staff and KIs**  **(N=22)** |
| --- | --- | --- |
| Female | 48 (100.0) | 15 (68.1) |
| Age | 21 (19-23) |  |
| At least secondary school education | 44 (91.7) |  |
| CES-D ≥10^3^ | 24 (50.0) |  |
| Prior PrEP use or participation in PrEP trial | 48 (100.0) |  |
| Adherent to PrEP^4^ | 20 (41.7) |  |
| Role in PrEP delivery  Key informant  Clinic staff member |  | 10 (45.5)  12 (54.5) |

PrEP=pre-exposure prophylaxis; AGYW=adolescent girl and young women; KI=key informants; CES-D=Center for Epidemiologic Studies Depression screening tool

^1^Gray shading indicates data either were not available in a given category or were not applicable

^2^Data are presented as medians and interquartile ranges (continuous data) or frequencies and percentages (categorical data)

^3^A CES-D score of 10 or greater is indicative of elevated depressive symptoms

^4^PrEP adherence among participants taking PrEP was measured using tenofovir diphosphate (TFV-DP) levels in dried blood spots. A TFV-DP level of 700 or more is indicative of 5 or more PrEP doses in about the month prior to sample collection and was used as the threshold for adherence.

**Supplemental Table 2.** Representative quotations from AGYW and KI workshops on key implementation barriers

| **Implementation barrier domain^1^** | **Theme** | **Representative quotation** |
| --- | --- | --- |
| ***AGYW Workshop Findings*** | | |
| Judgment taking PrEP or seeking mental health care | There is judgment and stigma around PrEP and mental health service seeking | *“My suggestion would be that we have a community dialogue with [AGYW] to break the stigma that lies in our community of not talking about issues that affect us mentally.”* |
| Poor service | AGYW feel they are mistreated, ignored, or disrespected and are concerned about confidentiality | *“Providers of PrEP at any institution have to be more friendly and respectful.”* |
| Time spent at clinic | AGYW do not want to spend a lot of time at the clinic or travel a large distance to get there | *“Looking at people back in the villages, some clinics are far, they have to travel, and they understand that it's always full when you get there, you would be there for the whole day, so it could be a barrier…to constantly go to the clinic”* |
| Trust issues | Parents and partners will be concerned about PrEP use and mental health issues | *“Another barrier is trust issues with parents, who will now begin to think you’re not taking care of yourself or you are seeking [mental health care] for attention.”* |
| Consistency of participation | AGYW may start sessions but will not continue with sessions regularly | *“It could be that people will respond, but the consistency will not be as expected, as we've seen with people taking medication and dropping before it finishes.”* |
| ***KI Workshop Findings*** | | |
| Accessibility | It is important to ensure appropriate location for the bench itself and to help make the clinic more accessible for AGYW; will need to ensure confidentiality and safety on the bench | *“The bench needs to be easily accessible. Like do adolescents need to take a taxi, a train-- you know what is the affordability of getting to the clinic and to access this sort of service?”* |
| Staff knowledge and ability | Counselors may not know where to refer patients and will not know about mental health counseling or the Friendship Bench | *“There are [training gaps] for the counselors who would be involved in the delivery of the intervention but more broadly, for the facility teams recognizing what this is all about and when they should be referring a patient who could potentially benefit from the intervention.”* |
| Stigma | There is a large amount of community, family, and facility-level stigma related to PrEP, HIV, and mental health issues | *“Mental health stigma is quite intense compared to other stigma. People would rather be HIV positive, COVID positive than to have poor mental health. So, I think that may prevent people to sometimes seek out mental health support.”* |
| Resources | Clinics are lacking staff (including counselors, social workers), referral points outside the clinic, and screening tools | *“We felt there was lack of staff, like social workers, psychologists and also referrals. When referring, the time and the availability of these professionals are not always in line with adolescents availability. Adolescents are in school and they might not be available when [referrals are] available so even if they get a referral the waiting list is really long.”* |
| AGYW needs | AGYW may not want to talk about mental health issues, they likely have other more pressing concerns (e.g., food insecurity), and do not want to spend a lot of time at the facility | *“There is the lack of basic needs of those seeking out clinic services. They wouldn't want to talk about mental health issues if they might focus on what am I going to eat tonight.”* |
